# Report on Operative Dentistry

**Published:** 1870-08

**Authors:** W. H. Morgan


### REPORT ON OPERATIVE DENTISTRY.

BY DR. W. H. MORGAN, D. D. S.

To the Southern Dental Association.

MR. PRESIDENT AND GENTLEMEN OF THE ASSOCIATION:—Your committee on Operative Dentistry would submit the following report:

I do not propose to go over the whole field of operative dentistry, including as it does, the operations of extracting, cleansing, pivoting and filling, but shall confine myself to the consideration mainly of the latter operation. There has been no great improvement in extracting teeth in the last few years of which I am aware, unless the less frequency of the operation may be considered such. The advancement made in other branches of operative dentistry, rendering this operation less a necessity than formerly—many teeth are now being saved permanently that were formerly removed. In the operation of cleansing, while there has been nothing new in the mode of accomplishing it, there has been an interest awakened upon this subject that betokens good, many practitioners now regarding it as a most important means of preserving the teeth, and of consequence in making the operation much more perfect than in former days. And yet I am persuaded very few operators, if any, ever perform this operation in the most perfect manner. Who ever cleansed and polished a set of teeth in the mouth of a patient, as he has done a single tooth with brush and lathe, or with the hand and such appliances as are in the hands or at the command of all? I affirm that until we so do it, we fall short in our duty as practitioners, and fail to give our patients the full benefit of our knowledge and capability. If this is true, are we not culpable? Do we not at least impliedly pledge our highest skill, our utmost knowledge to every patient who puts himself under our care, or who takes a seat in our operating chair?

Upon the subject of pivoting teeth, it is needless to dwell between cheap dental substitutes and the advances made in filling roots, and building up crowns; this operation is not nearly so often needed as in years past. And yet there are circumstances under which it is the operation, being the very best artificial tooth that can be worn.

The next subject I would call your attention to is the operation of caping the pulp, and thus attempting to preserve its vitality after exposure.

The most popular and successful treatment is the covering with *os artificiel*, and then filling over it with gold, after cutting away a sufficient portion of the *os artificiel* to enable the operator to make a good filling. This mode of practice has been adopted by many, after long years of experiment, in which every conceivable plan has been adopted to quiet this very sensative, and after exposure, intractable organ. Arching with gold foil, caping with gold plate, tin, lead, horn, wood, gutta percha, asbestos, oiled silk, vulcanite, risodontrophy, etc., etc., have all had their day and trial, running through a series of years. Each one of these has had its advocates, and all has been claimed for them that we could desire, but alas, they have each failed us in the hour of need, and for the want of higher attainments in knowledge and skill, we have been forced to sacrifice those valuable organs, and thus maim our patients for life. We trust a better and brighter day has dawned upon us, and that henceforth we will by the aid of *os artificiel*, be able to save where we were compelled to destroy. I can not say to whom the honor belongs of first using this material for this purpose. My attention was first called to it by our President in the summer of 1867. Since that time I have used it with the greatest satisfaction and success, rarely having any untoward effects following its use. It has been used by covering oiled or other silk or linen with it at about the consistency of thin cream, so that it would permeate the cloth, and then placing a small bit of the material thus prepared immediately over the exposed point of pulp, and then proceeding to fill the entire cavity with the *os artificiel*, and after hardening, removing a portion and filling with gold. Others following the example of our President, cover the exposure with collodion applied with a camels hair pencil, then fill. I would recommend the following mode of procedure: After thoroughly cleansing and otherwise preparing the cavity, with very delicate drills, I cut small retaining points as near the pulp as my judgment will permit me to approach, remove all foreign matter, wipe out with carbolic acid, dry with cotton or spunk, and put immediately upon the exposed point a small amount of *os artificiel*, in a very soft state, just sufficiently consistent to enable me to handle it; take up the superabundance of chloride of zinc with tissue paper, and then permit it to set for a few minutes. I then proceed to fill the entire cavity with the same material, as stiff as I can use it, over which I flow a little beeswax to protect from moisture, and let all remain until it sets properly. I prefer, if I can do so, to retain my patient a short time, thirty minutes is often sufficient, then remove a portion of my filling, fill with gold, thus completing the operation at a single sitting. I would not say that this was always advisable, for in some cases the cavity is so shallow and of consequence the amount of *os artificiel* to be left is so small, that it is desirable it should get as hard as possible before cutting away any portion, for fear of disturbing or removing that in contact with the pulp.

Such, has been my success by this mode of practice, that I think him, who does not try to preserve the vitality of exposed pulps by the use of *os artificiel*, a sinner above all other men.

Within the last year heavy gold foil has been attracting much more attention than in former times. I believe the credit of its introduction to the profession is due to Prof. Arthur, of Baltimore, some years since, perhaps about the time he first called attention to adhesive fillings. So many startling announcements have been made to the profession within the last few years, often unsettling all our preconceived notions and practice, that I have grown credulous with my years. I have so often been called upon and compelled by the almighty force of truth, to change to the right about, that I am getting rather used to it. Therefore, when I saw heavy foil so highly recommended, although persuaded that it would not answer, that it would prove a failure in the hands of a vast majority of practitioners, yet I determined to try it, and so compare with my own hands its merit with such as I had previously used. I had not gone as many had to the other extreme by using Nos. 2 and 3, but contented myself with 5 and 6, using mostly non-adhesive foils.

I ordered a few books of Nos. 15 and 20, and the first favorable opportunity took a strip of No. 15 and filled an approximal cavity in my usual mode. I was delighted. I next with the strips proceeded to fill a plain cavity, making an adhesive filling, using the mallet, and my assistant had not made a half dozen strokes, before I felt I had what I had been looking for for years. When the cavities are near the front of the mouth, I prefer using the strip, because of the facility with which I can control it, and the saving of time in introducing. If the cavities are far back in the mouth, and so more difficult of access, it is best to cut the gold in small pieces, say from one-quarter to one-half inch wide, and of various lengths, not exceeding an inch, and introduce them with pliers, heating each piece in all cases to a red heat, in a spirit lamp, before the introduction into the cavity.

Its advantages over light foil are many.

1st. It is softer.

2d. It does not harden so readily under the instrument.

3d. There is less danger of breaking thin walls in using it.

4th. It is more tractable, (if you will allow the phrase,) that is, it is more easily handled and controlled with the instrument.

5th. It is much more adhesive; its adhesiveness increasing with its thickness. I can not say in the exact ratio of the latter, nor do I pretend to know at what point in thickness this softness and adhesiveness will attain its maximum, but certainly its adhesiveness increases in the ratio of the increase of its softness, so that when we shall have determined at what thickness gold may be reduced to its softest state, we may, and probably will, have an exact gauge for its thickness, for our use.

6th. It finishes up when welded better than any other form of gold.

7th. The working of it gives a confidence to the operator, that is of incalculable value, inspiring him with zeal and enthusiasm that tend to fix a high ideal as to what his operations should be when finished. And thus he is led on and on, excelling himself and approaching perfection, the grand ultimatum of all our efforts. In the filing, cutting and polishing up, it is all the Dentist wants.

Now as to the force necessary to consolidate it. It can not, of course, take less than the lower numbers in detail, but it takes less force in the aggregate. It has been stated by some writers in the *Missouri Dental Journal* that higher than 20 could not be used with hand pressure. Yet, I often use No. 30 with hand pressure. It will not chap up when used with proper instruments, nor will the retaining surface be near so easily lost as with the lighter foils, unless from the effects of moisture, so marked is this quality that it will attract the attention of the most casual observer, perhaps the first time he uses it. It is much less inclined to double up upon itself when force is applied to it, than thin foil, this is in accordance with a mechanical law easily understood. The question is often asked, how heavy should a mallet be to use these heavy numbers? That is an unsettled question, different operators differing widest in their opinions; from one and one half to as high as nine ounces are used. I am inclined in this as in many other matters, to take medium ground and avoid both extremes, and prefer a mallet weighing from two to five ounces, finding the lighter best in some cases, and preferring the heavy in others. If we understood the laws of the physical forces and collisions clearly, we might then determine positively what weight of mallet would be best, leaving out of view the fact that it had to be used upon living organs, susceptible of pain. After we have so determined what weight of mallet will best or most perfectly accomplish the work we will have to learn, for this force should be distributed to give our patients the least pain, what weight of mallet and what force will accomplish our ends with the least pain to patients, and disturbance to the surrounding parts. I apprehend that the peculiarities of different cases will modify any rule we may fix upon, and that no positive data can ever be given for our government, and that we shall have to rely upon our judgment in each case as to how much force it will bear. We are all aware that some persons bear the effects of the mallet with much less suffering than others. The elasticity of the mallet, (the material of which it is made,) will often very greatly modify our procedure—the one being the most painful in one case, and the other in an other.

